# Evaluating immunogenicity of pathogen-derived T-cell epitopes to design a peptide-based smallpox vaccine

**DOI:** 10.1038/s41598-022-19679-3

**Published:** 2022-09-13

**Authors:** Huy Quang Quach, Inna G. Ovsyannikova, Gregory A. Poland, Richard B. Kennedy

**Affiliations:** Mayo Clinic Vaccine Research Group, Mayo Clinic, Rochester, MN 55905 USA

**Keywords:** Peptide vaccines, Protein vaccines

## Abstract

Despite the eradication in 1980, developing safe and effective smallpox vaccines remains an active area of research due to the recent outbreaks and the public health concern that smallpox viruses could be used as bioterrorism weapons. Identifying immunogenic peptides (epitopes) would create a foundation for the development of a robust peptide-based vaccine. We previously identified a library of naturally-processed, human leukocyte antigen class I-presented vaccinia-derived peptides from infected B cells. In the current study, we evaluated the immunogenicity of these T-cell peptides in both transgenic mouse models and human peripheral blood mononuclear cells. A vaccine based on four selected peptides provided 100% protection against a lethal viral challenge. In addition, responses from memory T cells remained unchanged up to five months. Our results validate a practical approach for identifying and verifying immunogenic peptides for vaccine development and highlight the potential of peptide-based vaccines for various infectious diseases.

## Introduction

Vaccination with vaccinia viruses (VACVs) of uncertain origins was directly responsible for the eradication of smallpox in 1980^1^, after which routine vaccination for smallpox ended. In science and public health, VACVs have remained an active area of research^2–5^ and have increasingly been used as vaccine vectors against various infectious pathogens including influenza, malaria, rabies, Zika^6–11^. Concerns have grown, however, about both the safety profile of these vaccinia-based vaccines and the re-emergence of vaccinia outbreaks in populations with a high risk of exposure to VACVs^12^. In addition, the 2001 anthrax attack in the US not only converted bioterrorism from a theoretical into realistic scenario, but also ignited a call of reintroduction of smallpox vaccines^13,14^. Therefore, vaccines and therapies for smallpox are still urgently needed.

Although the live, virus-based vaccines responsible for eradicating smallpox were very effective, they were also known to cause potentially serious, life-threatening side effects, had associated contraindications^15,16^. To improve the safety profile, newer generations of smallpox vaccines have been developed, but not deployed^17–19^. Researchers have recently sought to attenuate live VACVs and deliver them by microneedles^20,21^. Although safety profile improved with these attempts, public concerns about safety associated with live viruses is unlikely to have changed. An alternative approach that avoids the use of live viruses is therefore needed for the next generation of smallpox vaccines^22^.

Persons who received smallpox vaccines have been shown to have humoral (or B-cell) responses that correlated with protective levels of vaccinia infection, with a neutralizing antibody titer of less than 1:32 considered as high risk of infection^23^. In addition, cellular (or T-cell) responses have been reported as essential for viral clearance and recovery from infection^24^. Both B-cell and T-cell responses were persistent up to 50 or more years after smallpox vaccination^25,26^. More importantly, T cells, but not B cells, were shown as essential for containing local vaccinia replication^27^. Subsequently, numerous T-cell epitopes were identified by different approaches and showed their protective capacities at varying extents in vaccinated animals^28–31^. By using a mass spectrometry-based approach, we (IGO and GAP) identified 116 human leukocyte antigen (HLA) class I-restricted peptides derived from VACVs in infected B cells^32^. However, the immunogenicity of these naturally processed peptides remains unassessed. Herein, we evaluated the immunogenicity of these peptides in both transgenic mouse models and peripheral blood mononuclear cells (PBMCs) isolated from smallpox-vaccinated individuals. Of these 116 peptides, four peptides with the highest immunogenicity were selected, and a vaccine formulated with a pool of these four peptides provided 100% protection in immunized mice that were challenged with a lethal dose of VACVs.

## Results

### Screening peptide immunogenicity in mice

**Figure 1:**
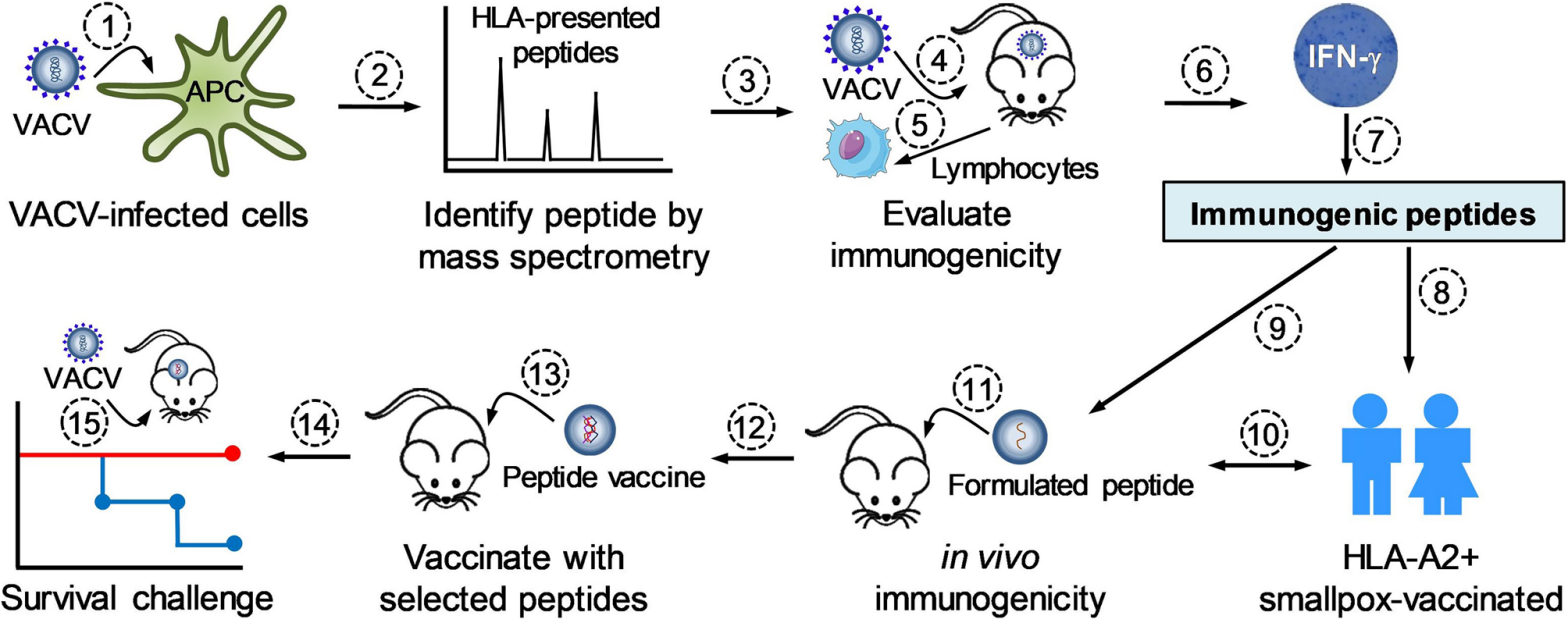
Procedure for identifying and evaluating of natural HLA class I-presented peptides. B cells were infected with NYCBOH strain of VACV (step 1) and vaccinia-derived HLA-presented peptides in infected B cells were purified by immunoaffinity chromatography followed by mass spectrometry analysis (step 2). Results from these two steps were reported in our previous publication^32^. The identified peptides were evaluated for immunogenicity using splenocytes and lymphocytes isolated from VACV-immunized mice by a mouse IFN-γ ELISPOT assay (step 3–7). Immunogenic peptides in VACV-immunized mice were verified in PBMCs isolated from smallpox-vaccinated individuals (step 8). These peptides were also formulated individually with IFA and evaluated for their immunogenicity in mice (step 9). Results from immunized mice and human PBMCs (step 10) helped to down-select peptides for a peptide-based vaccine (step 11 and 12), which was injected into mice (step 13). This peptide vaccine was validated for its protective activity in mice (step 14) upon a lethal viral challenge (step 15). *APC* antigen-presenting cell, *ELISPOT* enzyme-linked immunosorbent spot, *HLA* human leukocyte antigen, *IFA* incomplete Freund adjuvant, *IFN-γ* interferon-gamma, *MS* mass spectroscopy, *NYCBOH* New York City Board of Health, *PBMCs* peripheral blood mononuclear cells, *VACV* vaccinia virus.

**Figure 2:**
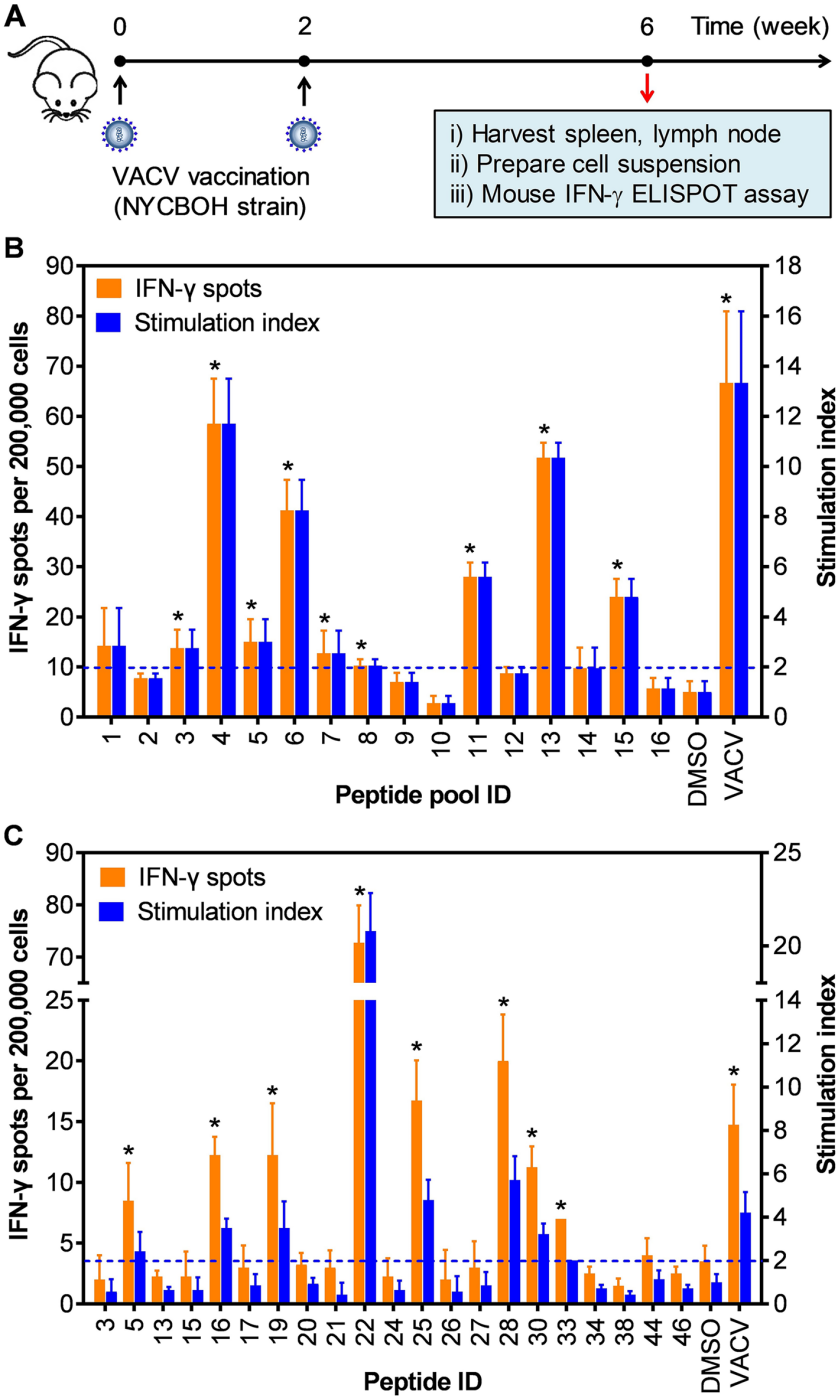
Representative results of evaluating immunogenicity of peptides in VACV-immunized B6.Cg-A2 mice. (**A**) Mice were vaccinated with VACV (NYCBOH strain) with a two-dose regimen, and splenocytes and lymphocytes were isolated four weeks after the second dose for the IFN-γ ELISPOT assay. (**B**) Immunogenicity of pooled peptides and (**C**) selected individual peptides were evaluated with splenocytes and lymphocytes from VACV-vaccinated B6.Cg-A2 mice. Positive responses were defined as those with a stimulation index ≥ 2 and *p* < 0.05. The asterisks (*) above each IFN-γ spot bar indicate positive peptide pools (**B**) or positive individual peptides (**C**). The dashed line represents a threshold of stimulation index for peptide selection. Each experiment condition was replicated four times, and data were presented as the mean ± the standard deviation (SD). DMSO and VACV were used as negative and positive controls, respectively.

The overall procedure is illustrated in Fig. 1. From a library of 116 peptides identified in our previous study^32^, we created 16 peptide pools by using the computer algorithm *Deconvolute This*, which was optimized for peptide matrix to identify T-cell antigens^33^. We then assessed the immunogenicity of these peptide pools in VACV-vaccinated mice. Because these peptides were identified as HLA-A2-restricted peptides, we evaluated their immunogenicity in three strains of transgenic mice carrying human HLA-A2 gene: C57BL/6-Tg(HLA-A2.1)1Enge/J (hereafter C57BL/6-A2), B6.Cg-Immp2lTg(HLA-A/H2-D)2Enge/J (hereafter B6.Cg-A2), and CB6F1-Tg(HLA-A*0201/H2-Kb)A*0201 (hereafter CB6F1-A2). The HLA-A2 mice were vaccinated with VACV (NYCBOH strain). One month after a booster vaccination, splenocytes and lymphocytes (hereafter lymphocytes) were isolated from vaccinated mice and used to evaluate peptide immunogenicity (Fig. 2A). We focused our evaluation on cellular immune responses induced by peptides with an interferon (IFN)-γ ELISPOT assay as a measure since HLA class I-presented peptides are unlikely to induce strong antibody responses^34,35^.

In mice, a positive IFN-γ response was defined as a response significantly higher (*p* < 0.05) than that induced by dimethylsulfoxide (DMSO, background control) with a stimulation index (i.e., fold increase over background) of ≥ 2. Presented as representative results in Fig. 2B, nine of 16 peptide pools (peptide pool ID #3, #4, #5, #6, #7, #8, #11, #13, and #15) induced positive IFN-γ responses of varying magnitude in B6.Cg-A2 mice. Of these positive peptide pools, pools #4 and #13 had the largest response, with approximately 55 spot-forming cells per 200,000 cells (Fig. 2B), which represented a stimulation index of > 10. Other positive peptide pools (peptide pool ID #3, #5, #6, #7, #8, #11, and #15) induced positive cellular responses of 9–41 IFN-γ spots per 200,000 cells with the stimulation index ranging from 2.1 to 8.3 (Fig. 2B). Lymphocytes from VACV-vaccinated mice could respond to the stimulation of peptide pools, confirming that at least one of these epitopes was presented during viral infection. Because of minor variations in cellular responses among the mice, only peptide pools that induced positive cellular responses in all three immunized mice were deconvoluted and tested individually for their immunogenicity.

From the positive peptide pools, *Deconvolute This* software identified 21 different peptides that potentially contributed to the observed peptide pool responses in VACV-immunized B6.Cg-A2 mice. However, only eight of 21 peptides (peptide ID #5, #16, #19, #22, #25, #28, #30, and #33) induced positive cellular responses in all immunized B6.Cg-A2 mice (Fig. 2C). Of these eight peptides, peptide #22 was the strongest inducer with 73 IFN-γ spots per 200,000 cells, a 20-fold increase over background. Interestingly, three peptides (peptide #22, #25, and #28) induced T-cell responses higher than VACV, likely reflecting the greater density of peptide/major histocompatibility complexes with soluble peptides compared with a virus that requires antigen processing and presentation. Other positive peptides (peptide #5, #16, #19, #30, and #33) moderately induced T-cell responses with the stimulation index of 2.0–5.7 (Fig. 2C).

We repeated the same screening procedure with C57BL/6-A2 and CB6F1-A2 mice and noted the difference in cellular responses among the three mouse strains. After two steps of screening the immunogenicity of peptide pools and individual peptides, five positive peptides were identified in C57BL/6-A2 mice, and seven positive peptides were identically identified in both B6.Cg-A2 and CB6F1-A2 mice (Table 1). Although the remaining peptides may still be useful in vaccine formulations destined for human use, they did not exhibit consistent immunogenicity in these HLA-A2 mouse models and were not considered further in the current study.

**Table 1:** Positive peptides in 3 different strains of female transgenic mice carrying human HLA-A2 gene.

ID	Sequence	Protein	Protein description	Protein/peptide homology	
				As compared to VARV	As compared to MPXV
**(A) Positive peptides in C57BL/6-A2 mice**					
1	ILIRGIINV	A ORF T	TNF receptor (CrmC)	Not present/-	Not present/–
7	LLISADDVQEIRV	A44L	Hydroxysteroid dehydrogenase	93.9%/100%	98.3%/100%
19	KIDYYIPYV	E2L (068)	Hypothetical protein	100%/100%	100%/100%
31	SLLFIPDIKL	K1L (043)	NF-kB inhibitor	96.8%/100%	95.8%/**F**LLFIPDIKL
33	GLLDRLYDL	O1L (079)	Hypothetical protein	99%/GLL**A**RLYDL	100%/100%
**(B) Positive peptides in B6.Cg-A2 and CB6F1-A2 mice**					
16	VLSLELPEV	D13L (127)	Viral coat protein	99.2%/100%	98.9%/100%
19	KIDYYIPYV	E2L (068)	Hypothetical protein	100%/100%	100%/100%
22	SLSNLDFRL	F11L (058)	Unknown function	95.8%/SLSNLDF**Y**L	97.2%/100%
25	ILMDNKGLGV	F1L (048)	Apoptosis inhibitor	83.1%/100%	84.6%/ILM**N**NKGLGV
28	ILDDNLYKV	G5R (091)	Viral morphogenesis	97%/100%	98.2%/100%
30	KLLLGELFFL	J3R (104)	Poly(A) polymerase	97.3%/100%	98.8%/100%
33	GLLDRLYDL	O1L (079)	Hypothetical protein	99%/GLL**A**RLYDL	100%/100%

Five (5) positive peptides were identified in C57BL/6-A2 mice model (A) while identical seven (7) positive peptides were identified in both B6.Cg-A2 and CB6F1-A2 mice (B). “Sequence” is the amino acid sequence based on the Copenhagen strain of vaccinia virus (VACV). “Protein” is the Copenhagen designation of the protein containing the indicated peptide. The number in parentheses is the designation for the ACAM2000 homolog. “Protein description” is obtained from www.poxvirus.org. The “Protein/peptide homology” columns list the sequence homology for the protein/peptide when compared to the sequences of Variola virus (VARV, India 1967 strain) and Monkeypox virus (MPXV, Zaire strain). Where differences occur, the VARV or MPXV sequences are listed with amino acid differences highlighted in bold and underlined.

The five peptides identified in C57BL/6-A2 mice derived from different proteins of NYCBOH strain of VACV (Table 1). However, two peptides (peptide ID #7 and #19) were identical with peptide sequences in variola virus (VARV, 1967 India strain) and monkeypox virus (MPXV, Zaire strain). The sequence of peptide #31 was identical with that in VARV, but different in one amino acid than MPXV. In contrast, peptide #33 had one amino acid difference from the VARV sequence, but it was identical to the MPXV sequence. Peptide #1 was identified in A ORF T protein of NYCBOH strain of VACV, but not in both VARV and MPXV. Similarly, seven peptides identified in B6.Cg-A2 and CB6F1-A2 mice were identical or nearly identical (differing by a single amino acid) to the VARV and MPXV homologues (Table 1).

### In vivo immunogenicity of selected individual peptides in mice

**Figure 3:**
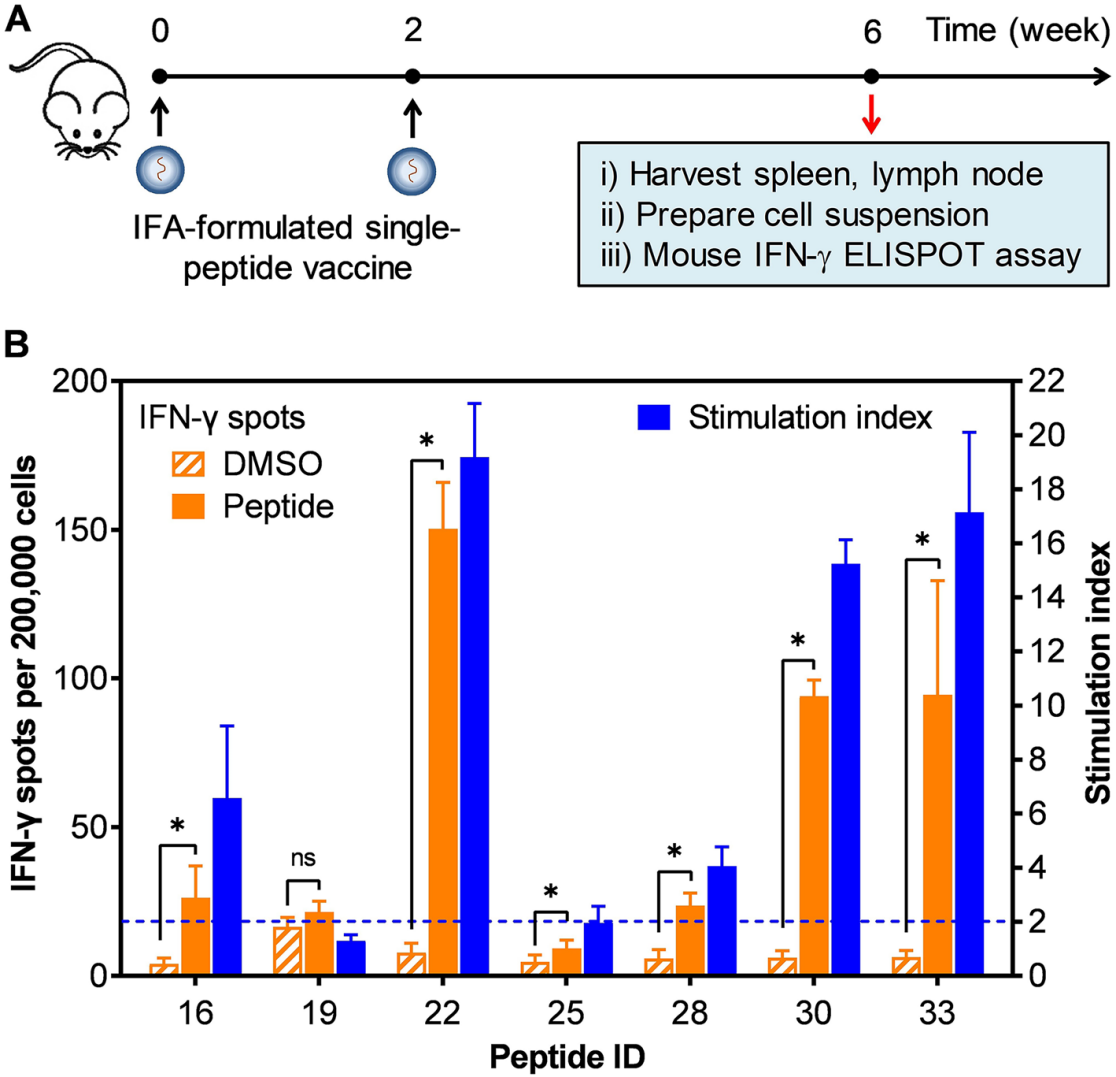
Evaluating immunogenicity of peptides in B6.Cg-A2 mice. (**A**) Each of seven selected individual peptides was formulated with IFA and injected into B6.Cg-A2 mice (n = 3). Four weeks after the booster vaccination, splenocytes and lymphocytes were isolated and used to evaluate the immunogenicity of individual peptides. (**B**) Isolated splenocytes and lymphocytes were stimulated with either DMSO (negative control) or corresponding peptides. The asterisks (*) indicate a significant difference (*p* < 0.05) in IFN-γ spots induced by individual peptides over DMSO background. Positive responses were defined as those with the stimulation index ≥ 2 and p < 0.05. The dashed line represents a threshold of selection for fold change. Each experiment condition was replicated four times and data were presented as mean ± standard deviation (SD).

Seven positive peptides (peptide #16, #19, #22, #25, #28, #30, and #33) were chosen for in vivo immunogenic evaluation in B6.Cg-A2 mice (Fig. 3A). Results from the IFN-γ ELISPOT assay showed that each of these seven peptides induced T-cell responses at varying degrees, with the stimulation index of 1.3–20 (Fig. 3B). Again, peptide #22 was the strongest inducer with approximately 150 IFN-γ spots per 200,000 cells, a 20-fold increase over the background, followed by peptides #33 and #30 with the stimulation index of 17 and 15, respectively. Peptides #25, #28, and #16 induced moderate responses, with the stimulation index ranging from 2 to 6.5 (Fig. 3B). Peptide #19 induced a nonsignificant response, with the stimulation index of 1.3.

### Recall immune response to peptides in smallpox-vaccinated subjects

**Figure 4:**
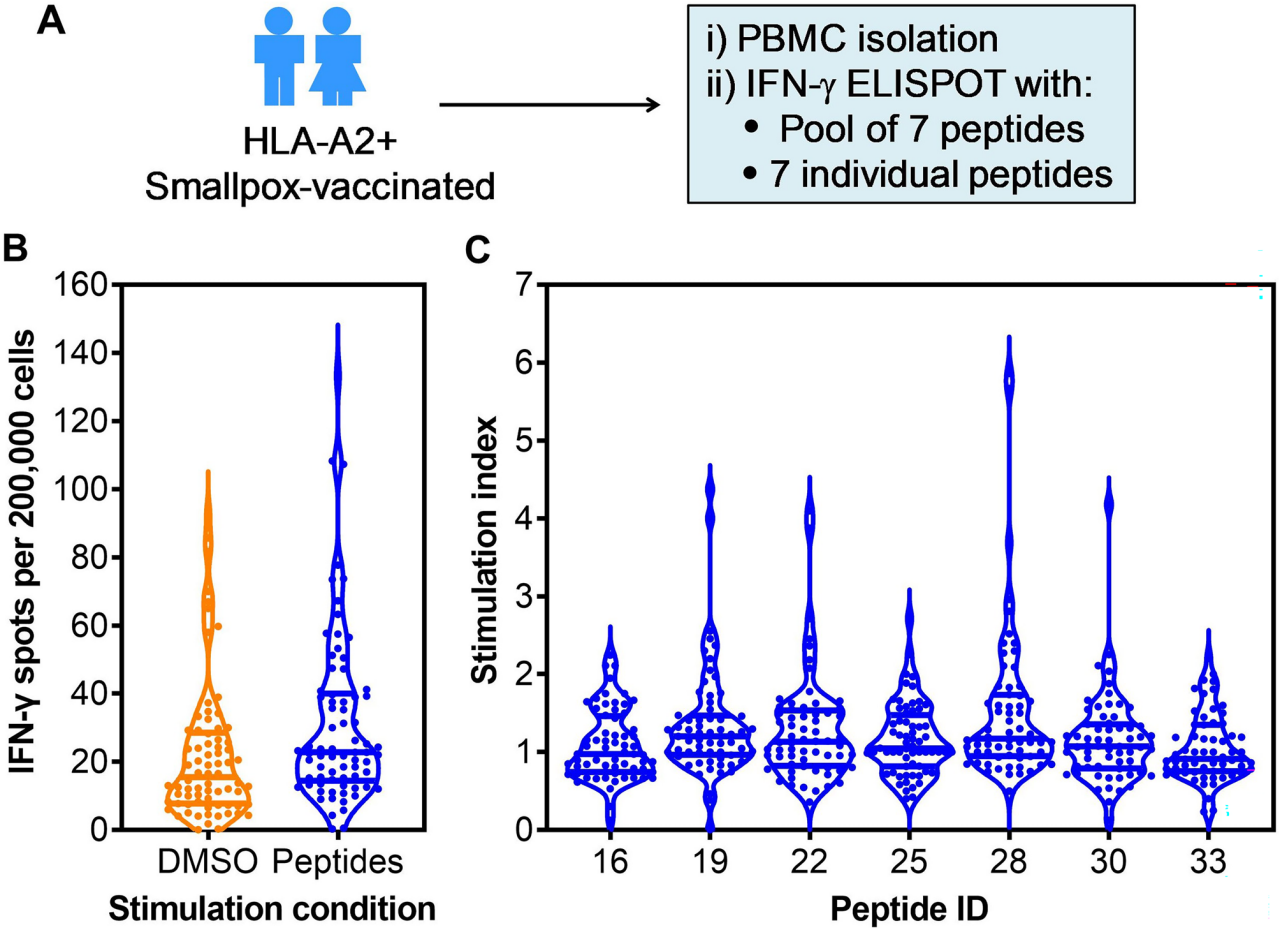
Immunogenicity of seven selected peptides in human PBMCs. (**A**) PBMCs were isolated from smallpox-vaccinated participants (n = 83) and used for a human IFN-γ ELISPOT assay. There were two cohorts of participant (n1 = 83, n2 = 64) recruited for the isolation of PBMCs used in IFN-γ ELISPOT assay with a pool of seven peptides (**B**) and seven individual peptides (**C**), respectively. DMSO was used as negative control. The experiment was replicated two times with PBMCs from each participant.

It was critical to ascertain which of these peptides were targeted by recall responses in vaccinated human population. Therefore, the immunogenicity of the seven peptides (peptide #16, #19, #22, #25, #28, #30, and #33) was also evaluated in PBMCs isolated from HLA-A2 supertype positive smallpox vaccine recipients. The PBMCs were incubated with either a pool of seven peptides or each of seven peptides overnight, and IFN-γ responses were evaluated (Fig. 4A). Due to a large variation of IFN-γ response in human PBMCs, a stimulation index of ≥ 1.5 and *p* < 0.05 was defined as a positive IFN-γ response. Although IFN-γ responses varied largely (Fig. 4B), the pool of seven peptides induced positive IFN-γ responses in PBMCs from 35 of 83 participants. For the participants with PBMCs that reacted to the peptide pool, the average stimulation index was 2.45, ranging from 1.55 to 6.17. These results confirmed that one or more of the seven peptides were recognized by PBMCs from smallpox-vaccinated participants.

Our next step was to ascertain the immunogenicity of each individual peptide in human PBMCs (Fig. 4C). Although all seven peptides induced detectable immune responses with the stimulation index of ≥ 1.5 in more than 10 of 64 participants, five peptides (peptide ID #19, #22, #25, #28, and #30) induced positive IFN-γ responses. Of these five peptides, peptide #28 induced positive IFN-γ responses in six participants with the highest stimulation index at 3.49 (Fig. 4C). Peptide #22, #25, and #30 induced positive IFN-γ responses in five, two, and three participants, respectively, with the stimulation index ranging from 2.07 to 2.73 (Fig. 4C). Peptide #19 had a stimulation index of 2.05 but induced positive IFN-γ responses in only one participant. Peptide #16 and #33 did not induce positive IFN-γ responses in PBMCs from tested participants. On the basis of the combined recall responses in human PBMCs (Fig. 4) and in vivo immunogenicity in mice (Fig. 3), four peptides (peptide #22, #25, #28, and #30) were selected for challenge study.

### Challenge study

**Figure 5:**
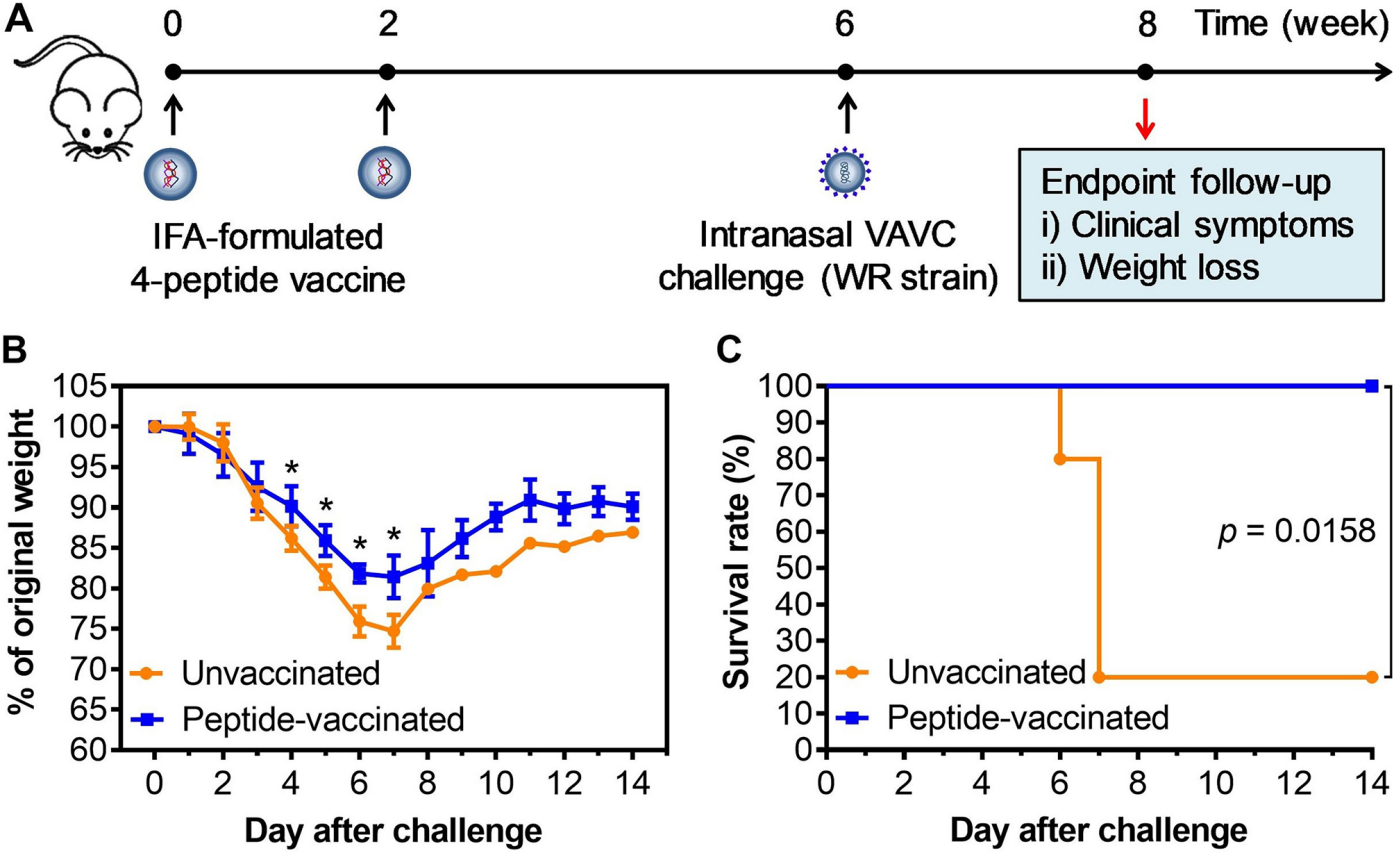
Survival of unvaccinated and peptide-vaccinated mice after lethal viral challenge. (**A**) Four selected peptides were formulated with IFA, and the mice were immunized with a two-dose regimen. Four weeks after the booster vaccination, the mice received an intranasal challenge with a lethal dose of VACV (WR strain). Clinical symptoms and weight loss were monitored daily for 2 weeks after viral challenge. (**B**) Weight of unvaccinated and peptide-vaccinated mice up to two weeks after the viral challenge. The asterisks (*) indicate a significant difference (p < 0.05) in weight between unvaccinated and peptide-vaccinated groups. Note that as of IACUC’s requirement, mice losing 25% of their weights were euthanized. After day 7, there was only one mouse in unvaccinated group; therefore, the difference in weight of mice between two groups were not statistically compared. (**C**) A vaccine consisting of four class I peptides (peptide #22, #25, #28, and #30) identified in this study conferred 100% protection in B6.Cg-A2 mice (n = 5).

Having verified that smallpox vaccine-induced recall responses recognized most of these peptides and that each peptide was immunogenic in mice, the next step was to determine whether or not a peptide-based vaccine could elicit protective immunity. A pool of four selected peptides (peptide ID #22, #25, #28, and #30) was formulated and immunized mice before a viral challenge (Fig. 5A). Although mice in both unvaccinated and peptide-vaccinated groups started to lose their weight from day 2 after the viral challenge, a significant difference (*p* < 0.05) in weight loss between unvaccinated and peptide-vaccinated mice was found from day 4 (Fig. 5B). A threshold of weight loss (> 25%) was found in unvaccinated mice at day 6. Peptide-vaccinated mice also experienced a similar pattern of weight loss but at moderate level and recovered from day 7 with none of them having weight loss of > 25% (Fig. 5B). Importantly, 100% of the peptide-vaccinated mice were protected against the lethal intranasal VACV challenge (Fig. 5C). In contrast, only 20% of unvaccinated mice survived to day 14, with 20% dying at day 6 and additional 60% dying at day 7. This observation suggested that immune responses induced by peptide-based vaccine can provide a robust protection against the lethal VACV infection.

### Long-term memory responses to peptide vaccination

**Figure 6:**
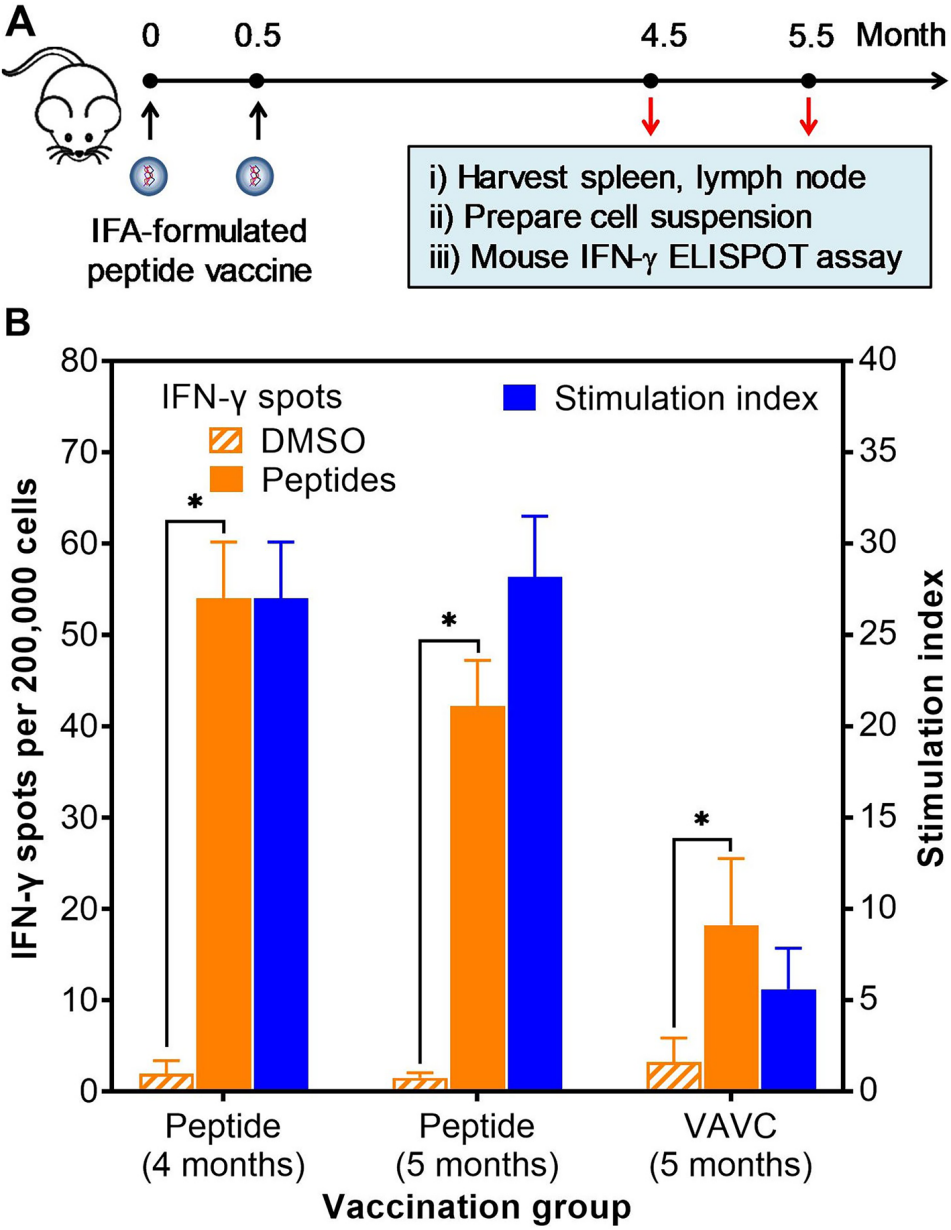
Long-term memory responses induced by peptide-based vaccine. (**A**) Mice received either the four-peptide/CpG/IFA vaccine or VAVC, as previously described. Four or five months after the booster vaccination, immune responses to peptide-pulsed target cells were tested using the mouse IFN-γ ELISPOT assay. (**B**) Murine cells were stimulated with either DMSO or a pool of four peptides (peptide #22, #25, #28, and #30). The asterisk (*) indicates a significant difference (p < 0.05) in IFN-γ spots induced by peptide over a DMSO background. Each experimental condition was replicated four times, and data were presented as mean ± standard deviation (SD).

To confirm whether the cellular responses induced by peptide vaccination elicit immunologic memory, mice were immunized with a pool of the four peptides (peptide #22, #25, #28, and #30) as described earlier and cellular responses were evaluated at four and five months after secondary immunization (Fig. 6A). At four months after the booster vaccination, peptide stimulation elicited a response of approximately 55 IFN-γ spots per 200,000 cells, with a stimulation index of 27 (Fig. 6B). Although the absolute number of IFN-γ spots decreased to approximately 43 per 200,000 cells at five months after the booster vaccination, the stimulation index even increased to 28 (Fig. 6B). These findings suggested that cellular responses induced by this peptide-based vaccine remained unchanged over the study period. At the same time, the stimulation index of VACV vaccine was 5.6, which was five times lower than that of the peptide-based vaccine (Fig. 6B).

## Discussion

Vaccination remains one of the best medical interventions to prevent infectious diseases, and characteristics of various vaccines continue to be refined to improve their safety, including antigen dose and antigen type. Thus, identifying pathogen-derived immunodominant epitopes is of paramount importance for developing successful protein, subunit, and peptide-based vaccines. Several approaches have used computer algorithm to predict immunodominant epitopes basing on genomic databases and identified peptides showed varying levels of immunogenicity and protective efficacy^28–31^. In the current study, we report a straightforward approach for evaluating peptide immunogenicity in both transgenic mouse models and human PBMCs. These peptides were shown to be HLA-A2 class I-restricted and were identified in VACV-infected B cells^32^. Similar to the peptides identified by others^28,31^, four peptides selected by this approach conferred full protection from viral challenge in immunized mice as well as induced long-term T-cell responses.

Although all three transgenic mouse models carried the human HLA-A2 gene, variations existed in the peptides identified as immunogenic in each mouse strain (Table 1), highlighting the importance of mouse model selection for peptide immunogenicity. The variations in peptides may reflect the variation in genetic background of three mouse strains^36^. The observed variation was slight: seven identical peptides were identified in both B6.Cg-A2 and CB6F1-A2 mice, and two of the five peptides (peptide #19 and #33) identified in C57BL/6-A2 mice overlapped with the seven peptides in B6.Cg-A2 and CB6F1-A2 mice (Table 1). Although the peptides used in this study were detected from human cells infected with the NYCBOH strain of VACV^32^, identical peptide sequences or sequences with just one amino acid difference from other poxviruses suggested that this approach can screen peptides used for a general vaccine against poxviruses. A recent study has evaluated a library of 20-mer peptides overlapping by 15 amino acids in E3L protein of VACV on human PBMCs and showed that a single amino acid change in peptide sequence across different strains of poxviruses was not sufficient to abrogate recognition by vaccinia-specific T cells^37^. More importantly, a pool of these seven peptides induced T-cell responses in PBMCs isolated from smallpox vaccine recipients, confirming that HLA-A2 transgenic mice are an appropriate model for screening peptides used for T-cell epitope-based vaccines^38^. One previous study compared the immunogenicity of VACV-derived T-cell peptides in human PBMCs and transgenic mice and showed peptide immunogenicity to be lower in PBMCs^38^. Similarly, our results showed a lower magnitude of IFN-γ responses upon incubation with the same peptide in PBMCs than in mice. For example, peptide #22 had a stimulation index of 20 in mice (Fig. 2C) but only 2.54 in human PBMCs (Fig. 4C). This decreased response may reflect differences in time since vaccination, with responses in mice being measured much closer to vaccination. This difference may also explain for the nonpositive responses of peptide #16 and #33 in human PBMCs since they were also among the least immunogenic peptides in mice, with the stimulation index of 3.5 and 2.05, respectively (Fig. 2C). These results thus highlight the difference in peptide immunogenicity in mice and human and reinforce the need to validate the peptide immunogenicity in the relevant host.

An extensive body of evidence indicates that cellular responses induced by peptide-based vaccine are sufficient to protect immunized animals from a lethal VACV challenge^28,31,39^. While our four selected peptides provided 100% protection in vaccinated mice (Fig. 5C), they did not prevent weight loss, indicating that protection against disease was incomplete. This might be due to weak or absent antibody responses. As our study focused on T cell responses, we did not measure VACV-specific antibodies in the immunized mice; therefore, we could not exclude the contribution of VACV-specific antibodies, if any, to the partial protection observed in immunized mice. HLA-restricted class I peptide immunization is unlikely to induce a robust antibody response, so we hypothesized that the protection observed was mainly mediated by T-cell responses. Our hypothesis is supported by 2 similar observations in 2 separate studies^27,39^. In their study on macaques, Gordon et al*.* found that the depletion of B cells had no effect on the size of skin lesions induced by the smallpox vaccine (Dryvax), but T-cell depletion resulted in progressive vaccinia, implying a critical role for T cells in viral clearance^27^. In the other study, Snyder et al*.* showed that a single T-cell peptide vaccine could provide full protection in HLA-A2 transgenic mice from intranasal VACV challenge, but the protection was eliminated with the depletion of T cells, suggesting that the protection was mainly mediated by T cells^39^.

Cellular responses have been maintained more than 50 years in smallpox vaccine recipients^25,26^, but that level of responsive persistence is in the context of immunization with a live-virus vaccine. We did not follow up cellular responses for such a long period in this study, but we found that cellular responses remained unchanged for five months (Fig. 6B). Although additional follow-up experiments are needed to evaluate longer-term memory responses, we expect that cellular responses induced by peptide-based vaccine will persist much longer. This hypothesis stems from our comparison that at the same time point (five months after the second dose), T-cell responses induced by our peptide vaccine were five times higher than the responses induced by live-virus vaccine (Fig. 6B). Further testing, however, is necessary to verify the longevity of immune responses induced by the peptide vaccine.

We validated an approach for identifying immunogenic peptides for peptide-based smallpox vaccines and evaluated T-cell responses induced by the selected peptides. This approach allowed our selected peptides to provide 100% protection in immunized mice against viral challenge. Many other factors, however, including administration route and adjuvants, could further improve the immunogenicity and protective activity of a peptide-based vaccine. For example, skin scarification with live VACV has been shown to be more effective in protecting mice against a lethal VACV challenge than other vaccination routes, such as intradermal, subcutaneous, or intramuscular routes^40^. In addition, incorporating an appropriate adjuvant could better drive T-cell responses, enhancing the protective activity of vaccines^41^. Although our study focused on identifying peptides derived from VACV, we strongly believe this approach could be adapted for other pathogens. In fact, the principle of this approach has been used to identify HLA peptides derived from influenza virus^42,43^, mycobacterium tuberculosis^44^, measles virus^45,46^, Zika virus^47^, SAR-CoV-2^48,49^.

In conclusion, we presented results for a highly effective approach to identify VACV-derived peptides and evaluate their immunogenicity in both mice and humans. In our challenge study, a peptide vaccine based on only four epitopes fully protected vaccinated mice. This vaccine also induced a long-term memory T-cell response. Our findings highlight the potential of peptide-based vaccines for human use.

## Methods

### Recruitment of study participants

This study was approved by the Mayo Clinic’s Institutional Review Board and performed under the Code of Ethics of the World Medical Association (Declaration of Helsinki). All participants provided their informed consent before blood draws. We recruited two cohorts (n1 = 83, n2 = 64) composed of 147 healthy participants with positive findings for HLA-A2 supertype who had received smallpox vaccine one to four years previously. Blood draws and biospecimen processing methods essentially identical to previously published protocols^50^. Whole blood was drawn from the participants by using Vacutainer CP tubes containing sodium citrate (BD Bioscience), and PBMCs were isolated and purified in accordance with manufacturer’s protocol. The purified PBMCs were then aliquoted in freezing media (RPMI medium supplemented with 20% heat-inactivated FCS and 10% DMSO) at 1 × 10^7^ cells/mL and stored in liquid nitrogen.

### Viruses

For this study, the New York City Board of Health (NYCBOH) strain of VACV was used to immunize mice and the Western Reserve (WR) strain of VACV was used for viral challenge. Both virus strains were grown in Hela S3 cells (#CCL-2.2, ATCC). Virus titer was quantified in Vero cells (#CCL-81, ATCC) and stored − 80 °C for future use. For some experiments, viruses were ultraviolet-inactivated, following a protocol published previously^25^.

### Mice

All experiments with mice were reviewed and approved by the Institutional Animal Care and Use Committee (IACUC) at Mayo Clinic. Three different transgenic mouse models carrying human HLA-A2.1 gene were tested: C57BL/6-Tg(HLA-A2.1)1Enge/J, strain #003475 (hereafter C57BL/6-A2); B6.Cg-Immp2lTg(HLA-A/H2-D)2Enge/J, strain #004191 (hereafter B6.Cg-A2) (The Jackson Laboratory); and CB6F1-Tg(HLA-A*0201/H2-Kb)A*0201, model #9659 (hereafter CB6F1-A2) (Taconic). The mice (all female) were maintained in a Biological Safety Level 2 containment facility under pathogen-free conditions at Mayo Clinic (Rochester, Minnesota) and used for the experiments when the mice were 6–12 weeks of age.

### Peptides and peptide pools

All peptides were synthesized at greater than 70% purity by Mimotopes Pty Ltd. Each peptide was assigned an ID number (peptide ID) when it was received from the supplier. Received peptides were dissolved in DMSO at 20 mg/mL. Peptide pools were created by combining equal concentrations of 16–20 peptides by using computer software specifically optimized for peptide matrix to identify T-cell antigens (*Deconvolute This*)^33^. With this software, each peptide was assigned to two different peptide pools and 16 peptide pools were created and assigned ID numbers (peptide pool ID) from 1 to 16.

### Screening peptide immunogenicity in mice

Three HLA-A2 mice were vaccinated by tail scratch with 1 × 10^6^ plaque-forming unit (pfu) VACV (NYCBOH strain). Mice were boosted with the same dose 14 days later. One month after the booster vaccination, the spleen and draining lymph nodes were isolated from vaccinated mice. A single cell suspension derived from the lymph nodes and spleen, hereafter referred to as lymphocytes, was obtained and used to screen the immunogenicity of peptide pools and then individual peptides using a mouse IFN-γ ELISPOT kit (catalog no. BD 551083, BD Biosciences). Briefly, 200,000 cells (suspended in 100 µL of cell culture medium RPMI) per well in a 96-well ELISPOT plate were incubated with 100 µL of RPMI medium containing 1 µg/mL (final concentration for each peptide) of either peptide pools or individual peptides. After incubation for 18 h in a 37 °C/5% CO_2_ incubator, plates were then washed, developed, allowed to dry in accordance with the manufacturer’s protocol. The dried plates were read on a CTL ELISPOT reader (Cellular Technology Limited). DMSO (at equal volume to the peptides) and inactivated VACV strain NYCBOH (multiplicity of infection = 2) in RPMI medium were used as negative and positive controls, respectively.

### Evaluating immunogenicity of selected peptides in mice

After the screening steps in VACV-immunized mice, seven peptides were selected, and their immunogenicities were evaluated individually in B6.Cg-A2 mice. Each of these peptides was formulated with incomplete Freund’s adjuvant (IFA) (catalog no. F5506, Sigma-Aldrich) using a rapid vortex method as reported previously^51^. Briefly, a 50:50 mixture of 100 µL of IFA and phosphate-buffered saline (PBS) solution containing 100 µg of CpG ODN 1826, 20 µg of individual peptide, and 140 µg of pan HLA-DR-binding epitope (PADRE) T helper (Th) peptide was vortexed at high speed for one hour to create a stable emulsion, which was then used for immunization. The mice (n = 3) were primed with 200 µL of peptide vaccine subcutaneously on the right flank and boosted with the same dose 14 days later. One month after the second dose, peptide immunogenicity was evaluated using the mouse IFN-γ ELISPOT assay, as described above.

### In vitro evaluation of peptide immunogenicity in smallpox-vaccinated participants

To complement the immunogenicity testing in mice described above, the seven selected peptides were also evaluated as a pool of seven peptides or as individual peptides for recall responses in PBMCs isolated from smallpox-vaccinated participants using a human IFN-γ ELISPOT kit (catalog no. 551849, BD Biosciences). The protocol for human IFN-γ ELISPOT assay was identical to that for the mouse IFN-γ ELISPOT assay described above, with the exception of using human PBMCs instead of murine lymphocytes. A pool of these seven peptides was tested with PBMCs from the 83 participants in cohort #1. Each selected peptide was also tested with PBMCs from the 64 vaccinated participants in cohort #2.

### Intranasal VACV challenge and survival experiments

Four peptides (peptide #22, #25, #28, and #30) were down-selected for survival challenge in B6.Cg-A2 mice. The mice (n = 5) were immunized twice (14 days apart) with a combination of four peptides (50 µg each) formulated with IFA, CpG, and PADRE Th peptide as described above. Four weeks after the booster vaccination, the mice were challenged by an intranasal inoculation with a lethal dose of 1 × 10^6^ pfu VACV (WR strain). Clinical symptoms and weight loss of challenged mice were monitored daily up to 14 days. Mice losing 25% of their body weights were euthanized in accordance with IACUC requirements.

### Long-term memory responses

Long-term memory responses were evaluated in B6.Cg-A2 mice (n = 3) that received the four-peptide (peptide #22, #25, #28, and #30) vaccine, as described above. The spleen and lymph nodes were isolated from vaccinated mice at four months or five months after the second dose of vaccine. T-cell responses were assessed using the mouse IFN-γ ELISPOT assay. As a control, a group of mice (n = 3) were vaccinated by tail scratch with 1 × 10^6^ pfu VACV (NYCBOH strain) as described above. Spleen and lymph node were isolated from vaccinia-vaccinated mice at five months after the second vaccine dose. T-cell responses from these mice were assessed in the same manner by using the mouse IFN-γ ELISPOT assay.

### Statistical analyses

The number of IFN-γ spots (calculated as spots per 200,000 cells—the number present in each well) from experimental groups were compared from that of negative control well (DMSO in cell culture RPMI). A two-tailed *t* test was used to compare the difference in the number of IFN-γ spots between groups. The stimulation index was defined as the average spot-forming cells in well stimulated with peptides divided by the average spot-forming cells in well stimulated with DMSO in cell culture RPMI (background). Positive responses in mice were defined as those with the stimulation index of ≥ 2 and a significance level of *p* < 0.05. Positive responses in human PBMCs were defined as those with the stimulation index of ≥ 1.5 and a significance level *p* < 0.05. All figures were plotted using GraphPad Prism 9.0 (GraphPad Software).

### Ethics statement

Animal experiments and all related experimental procedures were reviewed and approved by the Institutional Animal Care and Use Committee (IACUC) at Mayo Clinic. The animal experiments were conducted in accordance with the approved guidelines for animal experimentation at Mayo Clinic and the ARRIVE reporting guidelines. All human clinical study protocols were approved by the Mayo Clinic Institutional Review Board (IRB) and performed under the Code of Ethics of the World Medical Association (Declaration of Helsinki). All participants provided written informed consents in accordance with the Declaration of Helsinki.

## Data Availability

All data generated and analyzed for the current study are available from the corresponding author upon reasonable request.
